# A method for gaining a deeper insight into the aroma profile of olive oil

**DOI:** 10.1038/s41538-021-00098-z

**Published:** 2021-07-01

**Authors:** Daisuke Suzuki, Yuko Sato, Akane Mori, Hirotoshi Tamura

**Affiliations:** 1 Institute of Health Sciences, Ezaki Glico Co., Ltd., 4-6-5 Utajima, Nishiyodogawa-ku, Osaka, Japan; 2 The United Graduate School of Agricultural Sciences, Ehime University, 3-5-7 Tarumi, Matsuyama-shi, Ehime, Japan; 3 Faculty of Agriculture, Kagawa University, 2393 Ikenobe, Miki-cho, Kagawa, Japan

**Keywords:** Agriculture, Industry, Technology

## Abstract

Volatile compounds in food play a crucial role in affecting food quality and consumer preference, but the volatile compounds in olive oil are not fully understood due to the matrix effect of oil. The oiling-out assisted liquid–liquid extraction (OA-LLE), which we previously reported, is an effective method for isolating volatile compounds from edible oils with a strong matrix effect. However, when we apply OA-LLE to extra virgin olive oil (EVOO), the aromatic extracts contain non-volatile compounds such as pigments because of solvent-based extraction. Solvent-assisted flavor evaporation (SAFE) can remove such non-volatiles from extracts, but SAFE is affected by a matrix effect during distillation, resulting in a decrease in performance. By combining the advantages of OA-LLE and SAFE, we propose an effective approach, OA-LLE followed by SAFE (OA-LLE + SAFE), for extracting aroma compounds from EVOO. The “two assists” should help to better understand the native aroma profile of EVOO.

## Introduction

Olive oil, which is one of the most valuable and oldest oils, is extracted from olive fruit (*Olea europaea* L.). According to the International Olive Council (IOC), worldwide olive oil production has tripled in the last 60 years, reaching 3,379,000 t in the 2017/18 crop year. Major olive oil production is localized in the Mediterranean area, so the European Union (EU), especially Spain, Italy, and Greece, is the biggest producer. Recently, the production of olive oil has spread to other areas such as East Asia. When olive fruit is harvested at the appropriate ripening stage and is properly processed, olive oil with a unique flavor is produced.

Olive oil is mainly composed of glycerides, which account for >98% of the total composition^1^. The remaining minor fraction comprises volatile compounds, free fatty acids, phenols, tocopherols, pigments, sterols, waxes, hydrocarbons, and so on^2,3^. Volatile compounds in olive oil significantly influence the quality of the oil and hence consumer preference. The aroma profile of olive oil is known to be affected by many factors, including cultivar, olive fruit ripening stage, environment, extraction process (milling and malaxing, especially), and storage conditions^4–8^. These factors contribute to generating a wide variety and complexity of flavor of olive oils. Enzymatic reactions such as lipoxygenase (LOX, endogenous enzymes of olive tree) influence the formation of volatile compounds, which impart an olive oil-like note^9^, whereas unpleasant aroma compounds are generally formed by auto- and photo-oxidation. Therefore, numerous studies related to the aroma profile of olive oils have been conducted worldwide.

Understanding the native profile of the volatile compounds in olive oil is important for quality control and sustainable supply. For that, an effective method for extracting volatile compounds from olive oil is needed. We recently proposed a method for extracting a wide range of volatile compounds from edible oils and fat-enriched food based on the oiling-out effect^10,11^. The method, named the oiling-out assisted liquid–liquid extraction (OA-LLE), can be used to isolate the volatile compounds from the oil matrix, which has a strong matrix effect. Applying OA-LLE to only 5 g of coconut oil and dark chocolate resulted in the extraction of 44 and 54 aroma compounds, respectively. OA-LLE consists of two small-scale liquid–liquid extractions, making it easy to perform with no heating process. In addition, an organic solvent-based extraction is less susceptible to a strong matrix effect of triacylglycerols, thus resulting in increasing the efficiency of extracting the volatile compounds. On the other hand, when an organic solvent-based extraction is used on a sample containing a relatively large amount of non-volatile compounds such as pigments and phenolic compounds, some of the non-volatiles contaminate the aromatic extracts in some cases.

Solvent-assisted flavor evaporation (SAFE) proposed by Engel et al.^12^ has also been used to extract volatile compounds from olive oils^13–15^. SAFE can be used to isolate volatile compounds under mild conditions and to separate non-volatiles from aromatic extracts. However, SAFE is affected by the matrix effect of triacylglycerols during distillation, resulting in a decrease in performance. Considering the above, we focused on the advantages of OA-LLE and SAFE and hypothesize that OA-LLE followed by SAFE (OA-LLE + SAFE) can effectively extract volatile compounds from olive oil. That is, the dichloromethane layer of OA-LLE is charged into the SAFE apparatus as a sample solvent, and then SAFE is performed.

Thus, the aim of this study was to demonstrate an extraction method for a deeper understanding of the aroma compounds in olive oil. First, we performed OA-LLE and conventional methods, SAFE and head-space solid-phase micro extraction (HS-SPME),^16^ on extra virgin olive oil (EVOO). Next, to validate our hypothesis, OA-LLE + SAFE was performed, the extraction characteristics were investigated, and the results were compared with those obtained from the conventional methods. In addition, OA-LLE + SAFE was further applied using different types of EVOO. Our findings described in this study should contribute to gaining a deeper insight into the aroma profile of olive oil.

## Results

### Extraction of volatile compounds in EVOO using OA-LLE and conventional methods

**Fig. 1:**
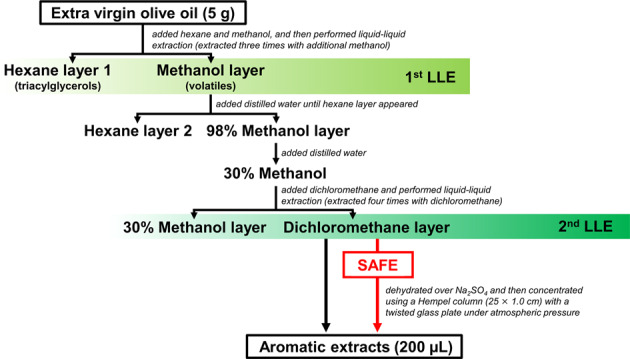
Procedures of OA-LLE with/without SAFE for EVOO. The procedures for OA-LLE and OA-LLE + SAFE are indicated by black arrows and a red arrow, respectively. The “Dichloromethane layer” contains most of the volatile compounds in EVOO. SAFE removed the non-volatiles such as pigments from the “Dichloromethane layer”.

The experimental plan is summarized in Table 1. Our previous study showed that OA-LLE is a powerful tool for isolating volatile compounds from edible oils.^11^ The extraction procedure for OA-LLE is shown in Fig. 1 (black arrows). The methanol layer in the first step of OA-LLE was diluted with distilled water to prepare a 30% methanol solution. This step is essential for performing liquid–liquid extraction with dichloromethane. From the results of the model study, 100% methanol was miscible with dichloromethane, but adding water to the methanol enables separation into two layers. Moreover, when the ratio of water was increased, the amount of methanol dissolved in the dichloromethane decreased significantly (Supplementary Table 1). This phenomenon allowed us to collect the volatile compounds in the dichloromethane layer. Consequently, 63 aroma compounds comprising 10 acids, 15 alcohols, 15 aldehydes, 10 esters, 1 furan, 6 hydrocarbons, 4 ketones, and 2 lactones were identified from only 5.0 g of EVOO using OA-LLE (Supplementary Table 2).

**Table 1:** Experimental plan for evaluating the extraction efficiency of volatile compounds in EVOO.

Method^a^	Cultivar	Country	Sample (g)	Aroma extracts (µL)	Repetition
OA-LLE	Hojiblanca	Spain	5.0	200	3
SAFE	Hojiblanca	Spain	5.0	200	3
HS-SPME	Hojiblanca	Spain	5.0	-	3
OA-LLE + SAFE	Hojiblanca	Spain	5.0	200	3
OA-LLE × 3 + SAFE	Hojiblanca	Spain	15.0	200	1
	Mission	Japan	15.0	200	1
	Lucca	Japan	15.0	200	1

^a^ *OA-LLE* oiling-out assisted liquid–liquid extraction, *SAFE* solvent-assisted flavor evaporation, *HS-SPME* head-space solid-phase micro extraction, *OA-LLE* *+* *SAFE* OA-LLE followed by SAFE, *OA-LLE* *×* *3* *+* *SAFE* the three dichloromethane layers separately extracted from 5.0 g of EVOO (15.0 g, in total) using OA-LLE were combined, the resulting combined dichloromethane layer was charged into a SAFE apparatus, and then distillation was conducted.

Among the volatile compounds, it has been reported that those responsible for the unique aroma of olive oil are mainly C5 and C6 aliphatic compounds^2^. These compounds were isolated from the oil matrix using OA-LLE. In addition, trace aroma compounds and semi-volatile compounds were also extracted. As a result, the removed layers, the hexane layers and 30% methanol layer, were sensorily judged to have no odor. In contrast, the OA-LLE extracts had a strong olive oil-like aroma.

These results indicate that most of the volatile compounds in the EVOO were in the “Dichloromethane layer”. However, the concentrated dichloromethane layer was dark green, which probably resulted from non-volatile compounds such as pigments (chlorophylls and carotenoids) (Supplementary Fig. 1a). In contrast, the extracts obtained using SAFE were colorless and transparent (Supplementary Fig. 1b). The number of aroma compounds isolated using SAFE and HS-SPME was 20 and 23, respectively (Table 2). The C5 and C6 aliphatic compounds were detected, indicating that these aroma compounds were successfully extracted.

**Table 2:** Volatile compounds identified in EVOO.

				OA-LLE + SAFE		SAFE		HS-SPME		
No.	RI	Volatile compound	CAS	Peak area^a^	µg/200 µL	Peak area	µg/200 µL	Peak area	Quantification^b^	Identification^c^
1	799	Octane	000111-65-9	0.0	0.0	0.0	0.0	3.6	–	RI, MS, Std
2	881	Ethyl acetate	000141-78-6	0.0	0.0	0.0	0.0	19.8	–	RI, MS
3	930	Ethanol	000064-17-5	0.0	0.0	0.0	0.0	265.7	–	RI, MS
4	970	3-Pentanone	000096-22-0	0.0	0.0	0.0	0.0	14.1	–	RI, MS
5	979	Methyl butanoate	000623-42-7	32.9	0.3	0.0	0.0	0.0	C	RI, MS
6	996	Decane	000124-18-5	0.0	0.0	26.8	0.5	19.3	A	RI, MS, Std
7	1013	1-Penten-3-one	001629-58-9	0.0	0.0	0.0	0.0	28.4	–	RI, MS
8	1031	Toluene	000108-88-3	40.9	0.5	0.0	0.0	0.0	Std	RI, MS, Std
9	1044	3-Hexanone	000589-38-8	39.4	0.8	0.0	0.0	0.0	B	RI, MS
10	1073	2-Hexanone	000591-78-6	62.8	1.2	0.0	0.0	0.0	Std	RI, MS, Std
11	1077	Hexanal	000066-25-1	0.0	0.0	17.0	0.5	9.0	G	RI, MS
12	1123	Ethylbenzene	000100-41-4	9.7	0.1	0.0	0.0	0.0	A	RI, MS
13	1137	(Z)-3-Hexenal	006789-80-6	0.0	0.0	13.9	0.4	11.2	G	RI, MS
14	1164	3-Penten-2-ol	001569-50-2	20.6	0.4	0.0	0.0	0.0	F	RI, MS
15	1171	2,6-Dimethyl-4-heptanone	000108-83-8	4.9	0.1	0.0	0.0	0.0	B	RI, MS
16	1189	3-Hexanol	000623-37-0	28.0	0.5	0.0	0.0	0.0	F	RI, MS
17	1200	Dodecane	000112-40-3	0.0	0.0	32.2	0.5	22.6	A	RI, MS, Std
18	1202	3-Methyl-1-butanol	000123-51-3	17.0	0.3	0.0	0.0	0.0	F	RI, MS
19	1215	(E)-2-Hexenal	006728-26-3	583.9	12.7	445.8	12.5	717.6	Std	RI, MS, Std
20	1243	β-Ocimene	013877-91-3	0.0	0.0	8.1	0.1	7.2	A	RI, MS
21	1269	Hexyl acetate	000142-92-7	48.1	0.5	28.5	0.4	34.2	Std	RI, MS, Std
22	1293	2,3-Dimethyl-1-butanol	019550-30-2	24.3	0.4	0.0	0.0	0.0	F	RI, MS
23	1314	(Z)-3-Hexenyl acetate	003681-71-8	363.8	4.0	292.4	4.2	392.7	Std	RI, MS, Std
24	1348	1-Hexanol	000111-27-3	115.9	2.0	64.6	1.4	42.9	F	RI, MS
25	1380	(Z)-3-Hexen-1-ol	000928-96-1	200.1	3.2	131.0	2.7	107.0	Std	RI, MS, Std
26	1392	Nonanal	000124-19-6	57.1	1.2	19.6	0.5	18.4	Std	RI, MS, Std
27	1402	(E)-2-Hexene-1-ol	000928-95-0	87.6	2.0	53.6	1.5	31.3	Std	RI, MS, Std
28	1440	Acetic acid	000064-19-7	11.2	0.6	37.0	2.7	465.5	Std	RI, MS, Std
29	1459	(E,Z)-2,4-Heptadienal	004313-02-4	7.3	0.1	0.0	0.0	0.0	D	RI, MS
30	1485	2-Ethyl-1-hexanol	000104-76-7	10.6	0.2	0.0	0.0	0.0	F	RI, MS
31	1499	Copaene	003856-25-5	25.4	0.3	19.6	0.3	0.0	A	RI, MS
32	1520	Benzaldehyde	000100-52-7	3.2	0.1	0.0	0.0	0.0	H	RI, MS
33	1553	1-Octanol	000111-87-5	16.0	0.3	0.0	0.0	0.0	F	RI, MS
34	1565	Dimethyl sulfoxide	000067-68-5	0.0	0.0	0.0	0.0	7.4	–	RI, MS
35	1615	Methyl benzoate	000093-58-3	11.5	0.1	5.2	0.1	0.0	I	RI, MS
36	1649	β-Farnesene	018794-84-8	14.0	0.2	0.0	0.0	0.0	A	RI, MS
37	1656	1-Nonanol	000143-08-8	11.4	0.2	0.0	0.0	0.0	F	RI, MS
38	1748	α-Farnesene	000502-61-4	230.8	3.0	44.9	0.8	14.6	A	RI, MS
39	1772	Methyl salicylate	000119-36-8	11.1	0.1	0.0	0.0	0.0	I	RI, MS
40	1837	Hexanoic acid	000142-62-1	20.0	0.4	0.0	0.0	0.0	Std	RI, MS, Std
41	1871	Benzyl alcohol	000100-51-6	21.2	0.3	5.8	0.1	3.8	J	RI, MS
42	1908	Phenylethyl alcohol	000060-12-8	53.5	0.7	14.3	0.2	10.0	J	RI, MS
43	1959	(E)-2-Hexenoic acid	013419-69-7	91.7	2.0	18.4	0.5	13.6	E	RI, MS
44	2036	(E)-Nerolidol	040716-66-3	107.7	0.8	0.0	0.0	0.0	K	RI, MS
45	2052	Octanoic acid	000124-07-2	13.3	0.2	0.0	0.0	0.0	Std	RI, MS, Std
46	2056	Dimethyl salicylate	000606-45-1	12.6	0.1	0.0	0.0	0.0	I	RI, MS
47	2076	Elemol	000639-99-6	34.8	0.4	0.0	0.0	0.0	J	RI, MS
48	2153	Nonanoic acid	000112-05-0	28.0	0.3	0.0	0.0	0.0	Std	RI, MS, Std
49	2172	4-Ethylphenol	000123-07-9	18.2	0.2	0.0	0.0	0.0	J	RI, MS
50	2216	Methyl palmitate	000112-39-0	5.8	0.1	0.0	0.0	0.0	I	RI, MS
51	2246	α-Cadinol	000481-34-5	87.3	1.1	23.3	0.4	0.0	J	RI, MS
52	2570	Vanillin	000121-33-5	55.5	1.3	0.0	0.0	0.0	H	RI, MS
Total				2639.1	43.3	1302.0	30.3	1956.7		

a × 10^5^. ^b^ Quantification: Std, authentic standard; A, toluene; B, 2-hexanone; C, hexyl acetate; D, 2,4-decadienal; E, hexanoic acid; F, 2-hexanol; G, *trans*-2-hexenal; H, *trans*-2-heptenal; I, ethyl decanoate; J, 2-phenoxyethanol; K, 1-hexadecanol. ^c^ Identification: *RI* retention index, *MS* mass spectral fragmentation pattern, *Std* authentic standard. This experiment was performed in triplicate and the means are presented.

### Extraction of volatile compounds in EVOO using OA-LLE followed by SAFE (OA-LLE + SAFE)

**Fig. 2:**
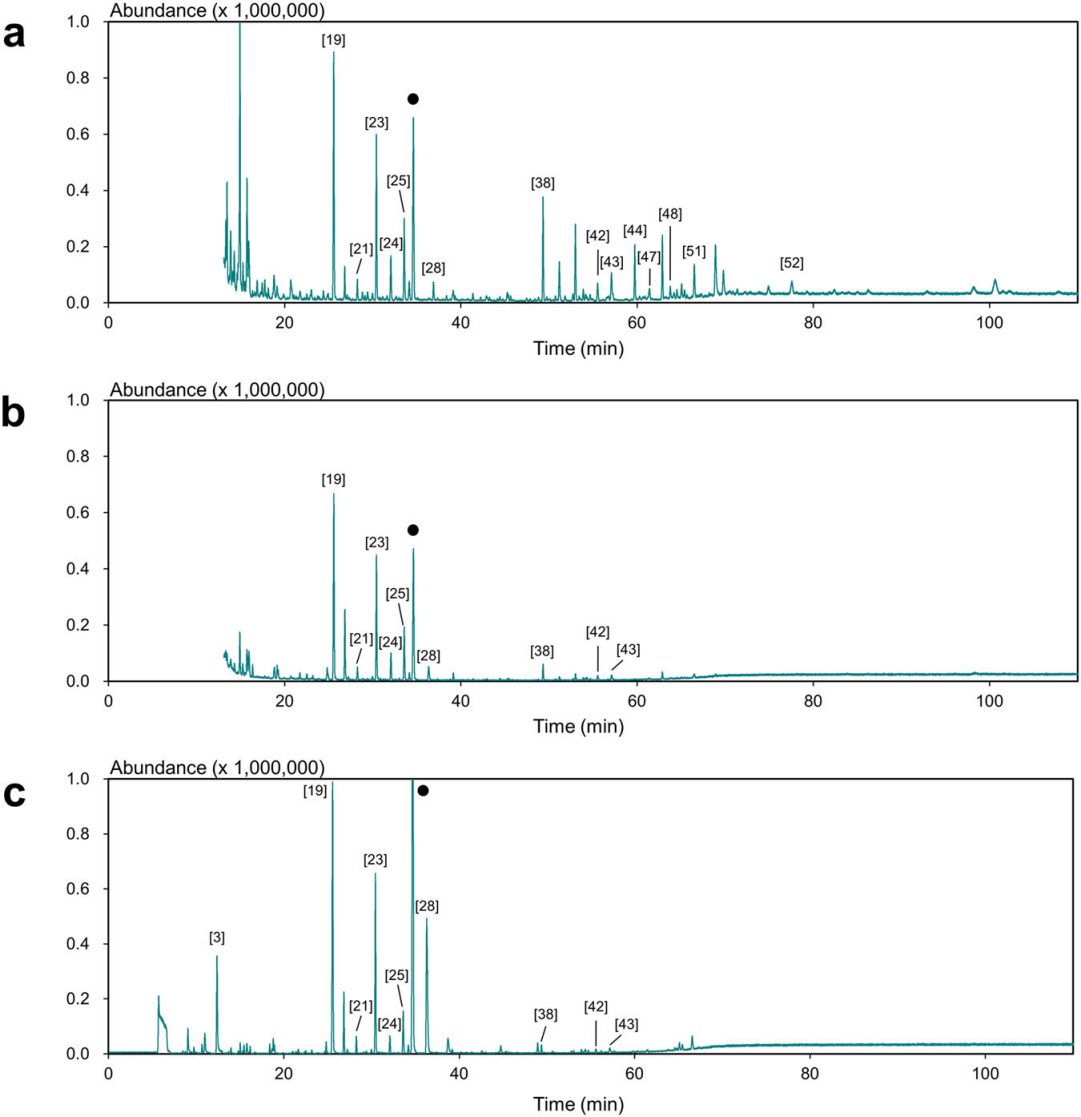
GC–MS chromatograms of the EVOO extracts obtained using each extraction method. The chromatograms for OA-LLE + SAFE, SAFE, and head-space solid-phase micro extraction (HS-SPME) are shown in **a**–**c**, respectively. Numbers refer to the volatile compounds listed in Table 2. The closed black circles (●) indicate the internal standard peaks (cyclohexanol).

The extraction procedure for OA-LLE + SAFE is shown in Fig. 1 (red arrow). In contrast to the OA-LLE extracts, the extracts obtained using OA-LLE + SAFE were colorless and transparent (Supplementary Fig. 1c). This result indicates that the non-volatiles contained in the dichloromethane layer were removed during SAFE. Moreover, 41 aroma compounds comprising 5 acids, 16 alcohols, 5 aldehydes, 7 esters, 5 hydrocarbons, and 3 ketones were extracted from 5.0 g of EVOO using OA-LLE + SAFE (Fig. 2 and Table 2). The C5 and C6 aliphatic compounds such as 3-penten-2-ol and (E)-2-hexenal were detected in the extracts obtained by OA-LLE + SAFE. It has been reported that C5 and C6 aliphatic compounds can be used as markers for ripening degree, geographic growing area condition, and quality-freshness, suggesting that OA-LLE + SAFE can be used for such studies.^17–19^

**Fig. 3:**
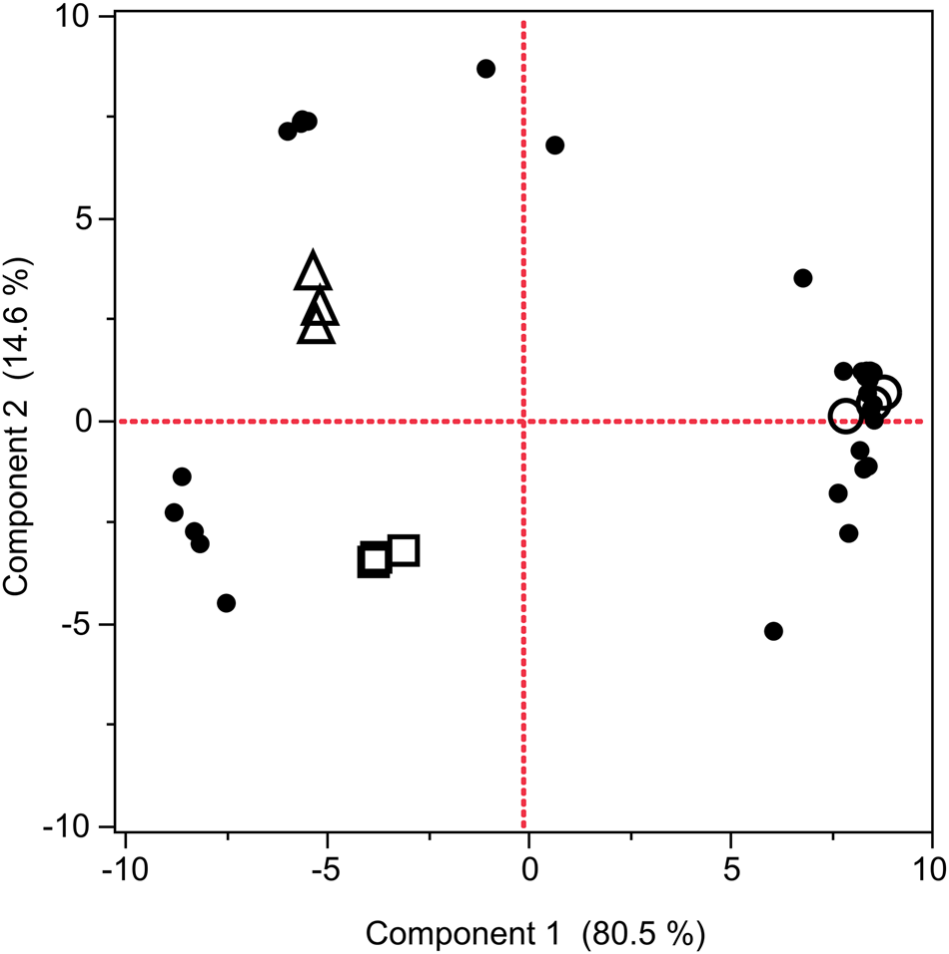
Principal component analysis (PCA) biplot of the aroma compounds in the EVOO. The peak areas of the aroma compounds were used to generate the PCA biplot. The number of aroma compounds obtained using the three methods, OA-LLE + SAFE, SAFE, and HS-SPME, was 41, 20, and 23, respectively. The closed black circles (●) indicate each aroma compound listed in Table 2, and the open circles (○), open squares (□), and open triangles (△) indicate OA-LLE + SAFE, SAFE, and HS-SPME, respectively. Each extraction was performed in triplicate.

In this study, OA-LLE + SAFE separated twice as many aroma compounds compared with the conventional methods. In addition, the total amounts of aroma compounds in the OA-LLE + SAFE extracts and the SAFE extracts were 43.3 ± 1.7 and 30.4 ± 6.6 µg/200 mL extract, respectively (*n* = 3, mean ± standard deviation (SD)). Principal component analysis (PCA) was applied to understand the extraction characteristics of each approach, and the PCA biplot is shown in Fig. 3. In applying PCA to the peak areas of each aroma compound obtained by using OA-LLE + SAFE, SAFE, and HS-SPME, the two principal components were able to explain 95.1% of the total variance. The biplot shows that OA-LLE + SAFE is located on the positive side on PC1 (80.5%). On the other hand, SAFE and HS-SPME were located on the negative side on PC1. HS-SPME is located on the positive side on PC2 (14.6%), while SAFE is located on the negative side on PC2. Correlation analysis revealed that PC1 and PC2 correlated with boiling point, *r* = 0.4157 (*p* < 0.05) and *r* = −0.3494 (*p* < 0.05), respectively. These results indicate that OA-LLE + SAFE can isolate not only the aroma compounds with a low boiling point but also the compounds with a relatively high boiling point. Comparing SAFE and HS-SPME, HS-SPME can isolate volatile compounds with a low boiling point better than SAFE because HS-SPME is a solvent-free method.

### Further application of OA-LLE + SAFE for a deeper insight into the aroma profile of EVOO

Applying OA-LLE + SAFE to EVOO overcomes the matrix effect of oil and removes the non-volatiles from the aromatic extracts. To obtain a deeper insight into the aroma profile of EVOO, we hypothesize that performing SAFE after several accumulations of the dichloromethane layer (OA-LLE) could further concentrate volatile compounds in the extracts. We performed OA-LLE three times (15.0 g of EVOO, in total) and then combined the dichloromethane layers. The volume of the sample solution was reduced to around 70 mL using the Hempel column with a twisted glass plate under atmospheric pressure (ca. 43 °C). After that, the semi-concentrated sample solution was charged into the SAFE apparatus (OA-LLE × 3 + SAFE).

**Fig. 4:**
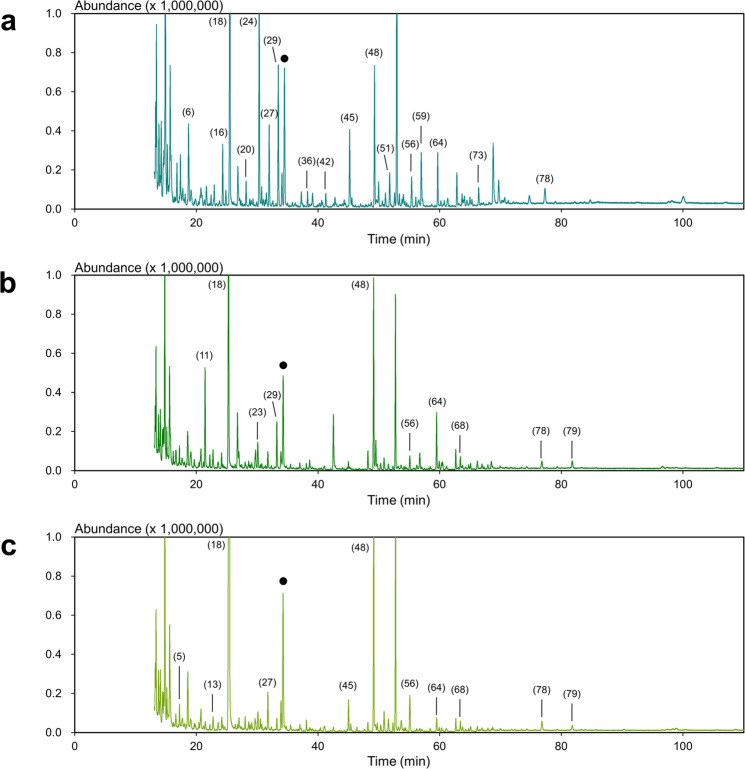
GC–MS chromatograms of the EVOO extracts obtained by OA-LLE × 3 + SAFE. The chromatograms for cv. Hojiblanca, cv. Mission, and cv. Lucca are shown in **a**–**c**, respectively. OA-LLE was performed three times separately (15.0 g of EVOO, in total), and the dichloromethane layers were combined. The sample solution was subjected to SAFE. Numbers refer to the volatile compounds listed in Supplementary Table 3. The closed black circles (●) indicate the peaks of the internal standard (0.005% (w/v) cyclohexanol).

We applied the OA-LLE × 3 + SAFE technique to three different types of EVOO, cv. Hojiblanca from Spain, cv. Mission from Japan, and cv. Lucca from Japan. As expected, the extracts obtained by using OA-LLE × 3 + SAFE were colorless and transparent, as were the extracts obtained with OA-LLE + SAFE. The GC–MS chromatograms obtained for these EVOOs are shown in Fig. 4. Compared with the result of OA-LLE + SAFE, the peak abundance of the volatile compounds was highly improved. The volatiles identified are listed in Supplementary Table 3.

**Fig. 5:**
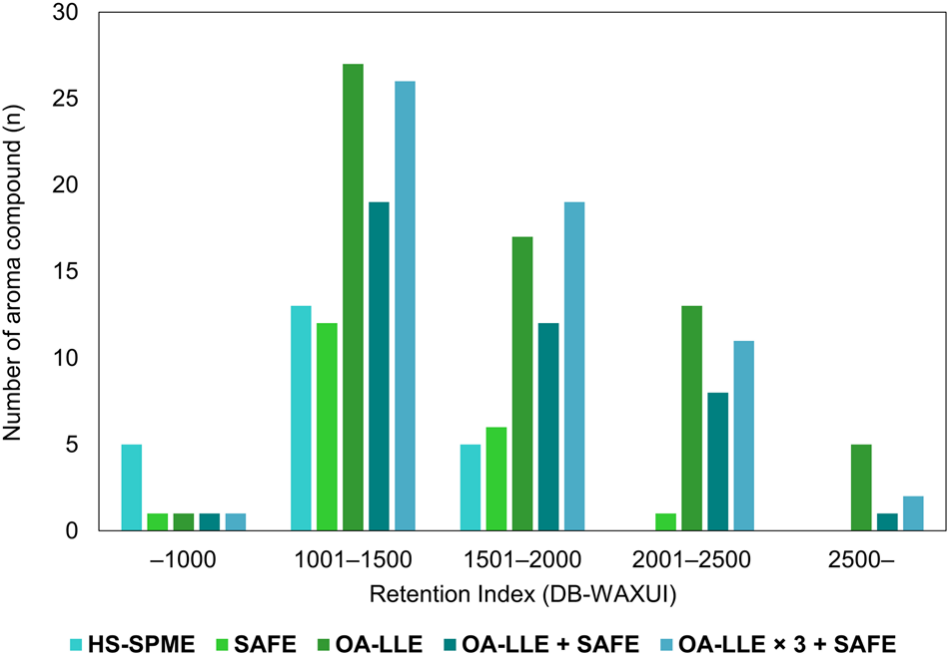
The number of aroma compounds obtained from the EVOO using each method. The number of aroma compounds obtained by HS-SPME, SAFE, OA-LLE, OA-LLE + SAFE, and OA-LLE × 3 + SAFE was 23, 20, 63, 41, and 59, respectively. The amount of EVOO (cv. Hojiblanca) used for the extractions was 15.0 g for OA-LLE × 3 + SAFE and 5.0 g each for the others. The combination of OA-LLE and SAFE improved the aroma extraction efficiency without non-volatiles appearing in the aromatic extracts. The aroma compounds exceeding RI 2000 were significantly extracted.

GC–MS analysis revealed that the number of aroma compounds in the extracts of Hojiblanca, Mission, and Lucca were 59, 45, and 39, respectively. In addition, the total amount of the aroma compounds in each extract (200 µL) was 145.1, 104.1, and 231.9 µg, respectively. The number of aroma compounds detected in each extract from Hojiblanca is summarized in Fig. 5. Many kinds of aroma compound were recovered using OA-LLE + SAFE and OA-LLE × 3 + SAFE. In particular, the aroma compounds exceeding RI 2000 dramatically improved.

## Discussion

To understand the native profile of aroma compounds in edible oil, we proposed OA-LLE in a previous paper^11^. However, some edible oils contain a relatively large amount of non-volatiles. The EVOO extracts obtained using OA-LLE were deep green (Supplementary Fig. 1a). It has been reported that olive oil contains pigments, and the content of phenolic compounds in olive oil is higher than that in other vegetable oils^20,21^. When we used OA-LLE on EVOO, volatile compounds and some non-volatiles were simultaneously isolated from triacylglycerols. Consequently, methanol is used to extract phenolic compounds from olive oil^22,23^.

Non-volatiles in the aroma extracts may contaminate a glass insert and a capillary column of GC–MS and may interfere with the gas chromatographic analysis, so it is desirable to remove non-volatiles from aromatic extracts as far as possible. To prevent the inclusion of non-volatiles in aromatic extracts, SAFE has been used on vegetable oils in recent studies^15,24^. The vegetable oils were diluted with dichloromethane and then charged into a SAFE apparatus. The non-volatile substances such as triacylglycerols and pigments were removed at this step. However, the volatile compounds with a low boiling point were mainly isolated from the vegetable oils due to a matrix effect of oil during SAFE. Our results also show that the aroma compounds with a low boiling point were mainly isolated using SAFE. Thus, an effective method for comprehensively extracting volatile compounds from EVOO needed to be investigated.

In this study, we hypothesize that OA-LLE + SAFE is an effective approach to isolating volatile compounds from EVOO. The dichloromethane layer of OA-LLE contains most of the volatile compounds in EVOO, and the triacylglycerols are removed. As shown in Supplementary Fig. 1c, the aroma extracts obtained using OA-LLE + SAFE were colorless, indicating that the non-volatiles were removed during distillation. Moreover, compared with the result from SAFE, the extraction efficiency was dramatically improved, indicating that the performance of SAFE was recovered. These results indicate that the strong matrix effect that occurs during the aroma extraction of EVOO has been overcome.

PCA revealed that SAFE and HS-SPME were located on the negative side on PC1, indicating that the aroma compounds with a low boiling point were mainly extracted (Fig. 3). Because HS-SPME is a solvent-free method, there is no solvent peak in the chromatogram, which means that HS-SPME is suitable for detecting volatile compounds with a low boiling point, compared with the other methods (Figs. 3 and 5). SAFE was also affected by the matrix effect of triacylglycerols, resulting in the extracts mainly consisting of volatile compounds with a low boiling point. On the other hand, OA-LLE + SAFE isolated 41 aroma compounds from 5.0 g of EVOO. Some semi-volatiles such as nonanoic acid and methyl palmitate were also recovered. These results indicate that OA-LLE is useful as a pretreatment for SAFE. This approach will also be helpful in extracting volatile compounds from other edible oils and its products, which contain a large amount of non-volatiles. Additionally, OA-LLE + SAFE may be suitable for analyzing rare or invaluable samples because this method requires only a small amount of sample^25^.

OA-LLE × 3 + SAFE can isolate not only volatile compounds but also trace aroma compounds and semi-volatile compounds with a relatively high boiling point. The extracts obtained using OA-LLE × 3 + SAFE were colorless and had quite a strong aroma. Using OA-LLE, it may be difficult to highly concentrate the aroma extracts because volatile compounds and non-volatiles will be concentrated simultaneously. Removing non-volatiles from the dichloromethane layer enables further concentration of volatile compounds to the extracts.

Many semi-volatile compounds and volatiles with a high affinity for triacylglycerols were found in the EVOO. Indeed, there were many kinds of aroma compound exceeding RI 2000 (Fig. 5). We have mainly focused on the C5 and C6 aliphatic compounds in EVOO so far. In this study we isolated additional potential aroma compounds in olive oil. To characterize the flavor property of olive oils, we should focus on not only volatile compounds but also semi-volatile compounds and compounds with high affinity for triacylglycerols. The results of this study indicate that OA-LLE × 3 + SAFE is an effective approach for deeply understanding an aroma profile of olive oil. Because the non-volatiles were eliminated by using SAFE, the extracts from OA-LLE can be accumulated many times, and the volatile compounds contained in olive oil can be highly concentrated. If necessary, the extracts can be further concentrated by performing three or more OA-LLE.

Detailed information on the chemical structure of the trace aroma compounds can be obtained by using the “two assists” method. As a result, we can improve the identification accuracy and can detect trace aroma compounds in olive oil. The trace aroma compounds may contribute to the diversity of aroma and flavor of olive oil. Combining the OA-LLE + SAFE technique with high-performance analytical instruments, e.g., GC×GC-TOF-MS, may give further insight into the volatile composition of olive oil^26,27^. Aroma-active compounds can be related with sensory attributes by means of odor activity value and pattern recognition techniques that use multivariate statistical analysis such as partial least square (PLS) algorithms, as has been done in previous studies^28,29^. These approaches allow us to objectively characterize the flavor profiles of olive oil. The aroma quality of olive oil is usually assessed by sensory evaluation, so detailed information on the aroma of olive oils may help that sensory evaluation. The effective extraction method described in this study should help with the quality control, improvement, and sustainable supply of olive oil.

## Methods

### Reagents and samples

Cyclohexanol (>98.0%) and *cis*-3-hexen-1-ol (97.0 + %) were obtained from Fujifilm Wako Pure Chemical Corporation (Osaka, Japan). Palmitic acid was acquired from Nacalai Tesque, Inc. (Kyoto, Japan). *trans*-2-Decenal (>93.0%), *trans*-2-heptenal (>95.0%), *trans*-2-hexen-1-ol (>95.0%), *trans*-2-hexenal (>97.0%), *cis*-3-hexenyl acetate (>97.0%), and hexyl acetate (>99.0%) were purchased from Tokyo Chemical Industry Co., Ltd. (Tokyo, Japan). The other reagents were described in our previous paper.^11^ The impurities derived from extraction solvents (hexane, methanol, and dichloromethane) for OA-LLE, SAFE, and HS-SPME were not observed. The monovarietal extra virgin olive oils (EVOO), Hojiblanca produced in Andalucía (Spain) and Mission and Lucca produced in Kagawa (Japan), were used in this study. These EVOOs were purchased from a market in Japan and stored at −20 °C until used.

### Model study for the 2^nd^ liquid–liquid extraction of oiling-out assisted liquid–liquid extraction (OA-LLE)

Dichloromethane (99.5%) and methanol (99.8%) were purchased from Fujifilm Wako Pure Chemical Corporation. The four methanol solutions (20, 30, 50, and 100%; 100 g each) were prepared separately and then liquid–liquid extraction was performed twice with 100 g of dichloromethane (200 g dichloromethane, in total). After that, the dichloromethane layers were dried with anhydrous sodium sulfate. Aliquots were subjected to gas chromatography with flame ionization detection (GC-FID) analysis, and the peak areas of methanol and dichloromethane were obtained. The methanol content in the dichloromethane was calculated using the ratio of these peak areas (peak area of methanol/peak areas of methanol and dichloromethane). The analytical condition was as follows: gas chromatograph, 6890 N GC System (Agilent Technologies, Inc., Santa Clara, CA, USA); column, DB-Wax (60 m × 0.25 mm i.d., 0.25 µm film thickness; Agilent Technologies, Inc.); carrier gas, He; make up gas, N_2_; flow rate, 1 mL min^−1^; oven temperature, 35 °C for 20 min, increased to 230 °C at 5 °C min^−1^, and finally maintained at 230 °C for 10 min; injection, split mode; injection volume, 0.2 µL; injection temperature, 200 °C; detector, FID; detection temperature, 250 °C. This experiment was conducted in quintuplicate.

### Oiling-out assisted liquid–liquid extraction (OA-LLE)

The experimental plan for evaluating the extraction efficiency of these methods is summarized in Table 1. The procedure for OA-LLE for EVOO is shown in Fig. 1 (black arrows). The extraction procedure was the same as in our previous report^11^. The extraction was performed in triplicate.

### Solvent-assisted flavor evaporation (SAFE)

In accordance with Peres et al.^30^, the following method was used with a minor modification. EVOO (5.0 g) and dichloromethane (10 mL) were transfered into a flask with a cap and then well mixed. Then the sample solution was charged into a SAFE apparatus. The extraction procedure was the same as in previous report^11^. The extraction was performed in triplicate.

### Head-space solid-phase micro extraction (HS-SPME)

A minor modification for HS-SPME was done according to a method described by Vichi et al.^31^. EVOO (5.0 g) was placed into a 20-mL glass serum vial with 1 µL of cyclohexanol as an internal standard. Using a laminated Teflon-silicone disc, the vial was subsequently screw-capped. The SPME fiber and the extraction procedure were same as in our previous work.^11^ The HS-SPME analysis was performed in triplicate.

### OA-LLE followed by SAFE (OA-LLE + SAFE)

As shown in Fig. 1 (red arrow), the “Dichloromethane layer” obtained by OA-LLE was charged into the SAFE apparatus. The procedure for SAFE is described above. The distillate was dried with 10.0 g of anhydrous sodium sulfate (stored overnight at −20 °C) and concentrated to 200 µL using a Hempel column (25 × 1.0 cm) with a twisted glass plate under atmospheric pressure (ca. 43 °C). The extraction was performed in triplicate. As a further application of OA-LLE + SAFE, OA-LLE was carried out three times separately, and the dichloromethane layers were combined. The sample solution was concentrated to around 70 mL and then subjected to SAFE. The distillate was concentrated to 200 µL using the same procedure described above. A 2 µL aliquot of these extracts was used for GC–MS analysis. This extraction was performed once for each sample.

### Gas chromatography–mass spectrometry (GC–MS)

The GC–MS system, the capillary column, and analytical conditions were same as those in our previous paper^11^. GC–MS was also conducted with a capillary column DB-1ms (30 m × 0.25 mm i.d., 0.25 µm film thickness; Agilent Technologies, Inc.) to isolate (E)-2-hexen-1-ol and cyclohexanol (internal standard) because the peaks of (E)-2-hexen-1-ol and cyclohexanol overlapped for DB-WAX Ultra Inert, showing a single peak. The ratio of peak areas obtained by DB-1ms was applied to the overlapped peaks obtained by DB-WAX Ultra Inert. The overlapped peak area was divided into the two peak areas, (E)-2-hexen-1-ol and cyclohexanol, and calculated. The sample (1 μL) was injected in the splitless mode. The GC oven was set at 30 °C for 2 min, increased to 230 °C at 3 °C min^−1^, and finally kept at 230 °C for 5 min.

### Identification and quantification of volatiles

Authentic standards, AromaOffice version 7.0 (Nishikawa Keisoku Co. Ltd., Tokyo, Japan), and AroChemBase version 7.0 (Alpha MOS, Toulouse, France) were used to identify the volatile compounds. The criteria of identification of the volatile compounds was same as in the previous report.^11^ For OA-LLE, SAFE, and OA-LLE + SAFE, the quantification was also performed in the same way as in the previous paper.^11^

### Chemical information

The boiling points of the volatile compounds were obtained from the PubChem and ChemSpider databases (last access date: 28 January 2020).

### Statistical analysis

All statistical analyses were carried out using Microsoft Excel version 15.0.4963.1000 and JMP 14.3 provided by SAS Institute Inc. (Cary, NC, USA). Principal component analysis (PCA) was performed to investigate the extraction characteristics of each method. PCA was applied to the peak area of the aroma compounds and was based on the correlation coefficient matrix. A biplot consisted of scores and loadings. Correlation analysis was used to evaluate the relationship between the loadings and the boiling point of the volatile compounds.

### Reporting summary

Further information on research design is available in the Nature Research Reporting Summary linked to this article.

## Supplementary information

Supplementary Information

Reporting Summary

## Data Availability

We declare that all the data supporting the findings of this study are available within this paper and its supplementary information.
