# Supplementary material for: A method for gaining a deeper insight into the aroma profile of olive oil
Source: NPJ Sci Food. 2021 Jul 1;5:16. doi: 10.1038/s41538-021-00098-z (PMC8249399; doi:10.1038/s41538-021-00098-z)
Supplement: Supplementary file 1 — Supplementary Information [file 41538_2021_98_MOESM1_ESM.pdf]

Supplementary information

A method for gaining a deeper insight into the aroma profile of olive oil

Daisuke Suzuki[1,2], Yuko Sato[1], Akane Mori[3], Hirotoshi Tamura[2,3*]

[1] Institute of Health Sciences, Ezaki Glico Co., Ltd., 4-6-5 Utajima, Nishiyodogawa-ku, Osaka, 555-8502, Japan
[2] The United Graduate School of Agricultural Sciences, Ehime University, 3-5-7 Tarumi, Matsuyama-shi, Ehime, 790-8566, Japan
[3] Faculty of Agriculture, Kagawa University, 2393 Ikenobe, Miki-cho, Kagawa, 761-0795, Japan
[*] Corresponding author (tamura.hirotoshi@kagawa-u.ac.jp)

Supplementary Table 1

Supplementary Table 2

References for Supplementary Table 2

Supplementary Table 3

Supplementary Fig. 1

Supplementary Table 1. Model study for the content of methanol in the dichloromethane layer of the 2nd liquid-liquid extraction of OA-LLE

Methanol concentration (%)	Methanol content in dichloromethane layer (%)
20	0.29 ± 0.08
30	1.42 ± 0.37
50	8.36 ± 0.67
100	mixed

This experiment was conducted in quintuplicate, and the means and standard deviations (SD) are presented (mean ± SD).

Supplementary Table 2. Volatile compounds identified in EVOO using OA-LLE

No.	RI	Volatile compound	CAS	Hojiblanca		Quantification[b]	Identification[c]
				Peak area[a]	μg/200 μL		
1	979	Methyl butanoate	000623-42-7	37.1	0.2	C	RI, MS
2	1033	Toluene	000108-88-3	76.7	0.6	Std	RI, MS, Std
3	1046	3-Hexanone	000589-38-8	258.6	3.2	B	RI, MS
4	1076	2-Hexanone	000591-78-6	465.1	5.8	Std	RI, MS, Std
5	1124	Ethylbenzene	000100-41-4	27.5	0.2	A	RI, MS
6	1154	1-Penten-3-ol	000616-25-1	26.5	0.3	F	RI, MS
7	1164	3-Penten-2-ol	001569-50-2	27.7	0.3	F	RI, MS
8	1182	Heptanal	000111-71-7	24.0	0.3	G	RI, MS
9	1192	3-Hexanol	000623-37-0	543.2	6.0	F	RI, MS
10	1200	Dodecane	000112-40-3	19.4	0.2	A	RI, MS
11	1216	(E)-2-Hexenal	006728-26-3	639.2	8.9	Std	RI, MS, Std
12	1217	2-Hexanol	000626-93-7	540.9	5.9	Std	RI, MS, Std
13	1229	2-Pentylfuran	003777-69-3	30.0	0.3	J	RI, MS
14	1246	1-Pentanol	000071-41-0	27.8	0.3	F	RI, MS
15	1255	Styrene	000100-42-5	12.0	0.1	A	RI, MS
16	1270	Hexyl acetate	000142-92-7	89.8	0.6	Std	RI, MS, Std
17	1281	1,2,4-Trimethylbenzene	000095-63-6	46.9	0.4	A	RI, MS
18	1287	Octanal	000124-13-0	49.8	0.7	L	RI, MS
19	1315	(Z)-3-Hexenyl acetate	003681-71-8	505.5	3.6	Std	RI, MS, Std
20	1323	(E)-2-Heptenal	018829-55-5	83.6	1.3	Std	RI, MS, Std
21	1336	6-Methyl-5-hepten-2-one	000110-93-0	20.5	0.3	B	RI, MS
22	1349	1-Hexanol	000111-27-3	165.7	1.8	F	RI, MS
23	1381	(Z)-3-Hexen-1-ol	000928-96-1	276.9	2.8	Std	RI, MS, Std
24	1393	Nonanal	000124-19-6	152.6	2.0	Std	RI, MS, Std
25	1403	(E)-2-Hexene-1-ol	000928-95-0	135.0	1.9	Std	RI, MS, Std
26	1430	(E)-2-Octenal	002548-87-0	75.5	1.0	L	RI, MS
27	1441	Acetic acid	000064-19-7	54.1	1.9	Std	RI, MS, Std
28	1463	(E,Z)-2,4-Heptadienal	004313-02-4	97.2	0.9	D	RI, MS
29	1525	Benzaldehyde	000100-52-7	11.5	0.2	H	RI, MS
30	1554	1-Octanol	000111-87-5	65.1	0.7	F	RI, MS
31	1627	Methyl benzoate	000093-58-3	25.6	0.1	I	RI, MS
32	1648	(E)-2-Decenal	003913-81-3	1087.4	12.9	Std	RI, MS, Std
33	1735	Citral	000141-27-5	53.7	0.6	N	RI, MS
34	1749	α-Farnesene	000502-61-4	338.9	2.9	A	RI, MS
35	1756	(E)-2-Undecenal	002463-77-6	147.1	1.7	N	RI, MS
36	1768	(E,Z)-2,4-Decadienal	025152-83-4	481.1	4.7	D	RI, MS
37	1785	Methyl salicylate	000119-36-8	18.4	0.1	I	RI, MS
38	1815	(E,E)-2,4-Decadienal	025152-84-5	684.5	6.6	Std	RI, MS, Std
39	1836	2,5-Hexanediol	002935-44-6	24.6	0.3	F	RI, MS
40	1838	Hexanoic acid	000142-62-1	48.2	0.7	Std	RI, MS, Std
41	1857	Nerylacetone	003879-26-3	44.6	0.3	M	RI, MS
42	1878	Benzyl alcohol	000100-51-6	107.3	0.9	J	RI, MS
43	1915	Phenylethyl alcohol	000060-12-8	84.0	0.7	J	RI, MS
44	1950	(E)-3-Hexenoic acid	001577-18-0	49.9	0.7	E	RI, MS
45	1960	(E)-2-Hexenoic acid	013419-69-7	184.3	2.6	E	RI, MS
46	2037	(E)-Nerolidol	040716-66-3	210.2	1.0	K	RI, MS
47	2054	Octanoic acid	000124-07-2	31.2	0.3	Std	RI, MS, Std
48	2072	Dimethyl salicylate	000606-45-1	22.1	0.1	I	RI, MS
49	2160	Nonanoic acid	000112-05-0	97.0	0.7	Std	RI, MS, Std
50	2211	δ-Cadinol	019435-97-3	56.6	0.4	J	RI, MS
51	2219	Methyl palmitate	000112-39-0	54.9	0.3	I	RI, MS
52	2247	α-Cadinol	000481-34-5	168.7	1.4	J	RI, MS
53	2256	Ethyl palmitate	000628-97-7	71.9	0.4	I	RI, MS
54	2266	Decanoic acid	000334-48-5	45.9	0.4	Std	RI, MS, Std
55	2273	(E,E)-Farnesal	000502-67-0	84.7	0.8	D	RI, MS
56	2405	Methyl jasmonate	001211-29-6	109.3	0.6	I	RI, MS
57	2478	Dodecanoic acid	000143-07-7	131.9	0.9	Std	RI, MS, Std

Supplementary Table 2. (*continued*)

No.	RI	Volatile compound	CAS	Hojiblanca Peak area[a]	Hojiblanca μg/200 μL	Quantification[b]	Identification[c]
58	2484	Ethyl oleate	000111-62-6	222.7	1.3	I	RI, MS
59	2571	Vanillin	000121-33-5	245.5	3.7	H	RI, MS
60	2672	δ-Tetradecalactone	002721-22-4	249.6	2.3	Std	RI, MS, Std
61	2690	Tetradecanoic acid	000544-63-8	170.9	1.2	Std	RI, MS, Std
62	2893	δ-Hexadecalactone	007370-44-7	318.9	2.2	Std	RI[d], MS[e], Std
63	2907	Hexadecanoic acid	000057-10-3	8042.9	43.8	Std	RI, MS, Std
Total				18299.9	150.3		

[a] $\times 10^5$. [b] Quantification: Std, authentic standard; A, toluene; B, 2-hexanone; C, hexyl acetate; D, 2,4-decadienal; E, hexanoic acid; F, 2-hexanol; G, *trans*-2-hexenal; H, *trans*-2-heptenal; I, ethyl decanoate; J, 2-phenoxyethanol; K, 1-hexadecanol; L, nonanal; M, 2-undecanone; N, *trans*-2-decenal. [c] Identification: RI, retention index; MS, mass spectral fragmentation pattern; Std, authentic standard. [d] Ref. S1. [e] Ref. S2. This experiment was performed in triplicate and the means are presented.

References for Supplementary Table 2.

S1. High, R., Bremer, P., Kebede, B. & Eyres, G. T. Comparison of four extraction techniques for the evaluation of volatile compounds in spray-dried New Zealand sheep milk. *Molecules* **24**, 1917 (2019).

S2. Schlutt, B., Moran, N., Schieberle, P. & Hofmann, T. Sensory-directed identification of creaminess-enhancing volatiles and semivolatiles in full-fat cream. *J. Agric. Food Chem.* **55**, 9634–9645 (2007).

Supplementary Table 3. Volatile compounds identified in EVOOs using OA-LLE × 3 + SAFE

No.	RI	Volatile compound	CAS	Hojiblanca Peak area[a]	Hojiblanca µg/200 µL	Mission Peak area	Mission µg/200 µL	Lucca Peak area	Lucca µg/200 µL	Quantification[b]	Identification[c]
1	979	Methyl butanoate	000623-42-7	82.3	0.9	0.0	0.0	0.0	0.0	C	RI, MS
2	979	3-Methyl-3-buten-2-one	000814-78-8	0.0	0.0	178.8	4.3	0.0	0.0	B	RI, MS
3	1028	2-Methyl-3-buten-2-ol	000115-18-4	0.0	0.0	142.4	3.0	0.0	0.0	F	RI, MS
4	1031	Toluene	000108-88-3	139.5	2.0	0.0	0.0	119.0	1.9	Std	RI, MS, Std
5	1044	3-Hexanone	000589-38-8	178.3	3.7	110.5	2.7	129.0	3.0	B	RI, MS
6	1073	2-Hexanone	000591-78-6	341.9	7.0	192.6	4.6	0.0	0.0	Std	RI, MS, Std
7	1077	Hexanal	000066-25-1	0.0	0.0	0.0	0.0	295.9	7.9	G	RI, MS
8	1098	Undecane	001120-21-4	0.0	0.0	0.0	0.0	38.4	0.6	A	RI, MS
9	1123	Ethylbenzene	000100-41-4	38.4	0.5	0.0	0.0	0.0	0.0	A	RI, MS
10	1131	*p*-Xylene	000106-42-3	0.0	0.0	0.0	0.0	51.6	0.8	A	RI, MS
11	1137	(Z)-3-Hexenal	006789-80-6	0.0	0.0	357.5	9.8	0.0	0.0	G	RI, MS
12	1150	1-Penten-3-ol	000616-25-1	36.8	0.7	58.7	1.3	0.0	0.0	F	RI, MS
13	1164	3-Penten-2-ol	001569-50-2	68.5	1.2	89.5	1.9	75.0	1.6	F	RI, MS
14	1171	2,6-Dimethyl-4-heptanone	000108-83-8	8.3	0.2	0.0	0.0	0.0	0.0	B	RI, MS
15	1174	*o*-Xylene	000095-47-6	0.0	0.0	0.0	0.0	52.5	0.8	A	RI, MS
16	1189	3-Hexanol	000623-37-0	210.2	3.8	66.2	1.4	72.9	1.5	F	RI, MS
17	1202	3-Methyl-1-butanol	000123-51-3	69.3	1.3	17.9	0.4	0.0	0.0	F	RI, MS
18	1215	(E)-2-Hexenal	006728-26-3	1720.3	40.1	1336.2	36.5	6099.9	162.6	Std	RI, MS, Std
19	1243	β-Ocimene	013877-91-3	0.0	0.0	82.7	1.4	70.5	1.1	A	RI, MS
20	1269	Hexyl acetate	000142-92-7	88.9	1.0	0.0	0.0	70.7	0.9	Std	RI, MS, Std
21	1284	Octanal	000124-13-0	31.8	0.7	21.6	0.6	27.5	0.7	L	RI, MS
22	1293	2,3-Dimethyl-1-butanol	019550-30-2	0.0	0.0	31.4	0.7	0.0	0.0	F	RI, MS
23	1306	(Z)-2-Penten-1-ol	001576-95-0	0.0	0.0	123.4	2.6	98.3	2.1	F	RI, MS
24	1314	(Z)-3-Hexenyl acetate	003681-71-8	916.6	10.8	0.0	0.0	0.0	0.0	Std	RI, MS, Std
25	1319	(E)-2-Heptenal	018829-55-5	74.3	1.9	0.0	0.0	62.1	1.8	Std	RI, MS, Std
26	1331	6-Methyl-5-hepten-2-one	000110-93-0	22.1	0.5	0.0	0.0	0.0	0.0	B	RI, MS
27	1348	1-Hexanol	000111-27-3	295.7	5.4	81.6	1.7	160.6	3.3	F	RI, MS
28	1353	4-Hydroxy-4-methyl-2-pentanone	000123-42-2	0.0	0.0	22.2	0.5	0.0	0.0	B	RI, MS
29	1380	(Z)-3-Hexen-1-ol	000928-96-1	516.2	8.8	190.1	3.8	50.5	1.0	Std	RI, MS, Std
30	1392	Nonanal	000124-19-6	140.3	3.1	0.0	0.0	123.1	3.1	Std	RI, MS, Std
31	1402	(E)-2-Hexene-1-ol	000928-95-0	235.7	5.6	50.6	1.4	245.3	6.7	Std	RI, MS, Std
32	1426	(E)-2-Octenal	002548-87-0	23.8	0.5	0.0	0.0	0.0	0.0	L	RI, MS
33	1440	Acetic acid	000064-19-7	25.7	1.6	12.7	0.9	0.0	0.0	Std	RI, MS, Std
34	1447	1-Heptanol	000111-70-6	4.4	0.1	0.0	0.0	0.0	0.0	F	RI, MS
35	1459	(E,Z)-2,4-Heptadienal	004313-02-4	57.4	0.9	35.0	0.6	28.4	0.5	D	RI, MS
36	1485	2-Ethyl-1-hexanol	000104-76-7	54.5	1.0	23.2	0.5	39.0	0.8	F	RI, MS
37	1489	(E,E)-2,4-Heptadienal	004313-03-5	15.8	0.2	0.0	0.0	0.0	0.0	D	RI, MS
38	1499	Copaene	003856-25-5	68.8	1.0	0.0	0.0	0.0	0.0	A	RI, MS
39	1509	3,5-Octadien-2-one	038284-27-4	0.0	0.0	4.9	0.1	0.0	0.0	B	RI, MS
40	1520	Benzaldehyde	000100-52-7	11.3	0.3	0.0	0.0	0.0	0.0	H	RI, MS
41	1534	Linalool	000078-70-6	0.0	0.0	6.9	0.1	0.0	0.0	F	RI, MS
42	1553	1-Octanol	000111-87-5	59.1	1.1	0.0	0.0	21.2	0.4	F	RI, MS
43	1615	Methyl benzoate	000093-58-3	34.3	0.3	14.1	0.2	0.0	0.0	I	RI, MS
44	1634	L-(-)-Menthol	002216-51-5	0.0	0.0	11.2	0.2	10.6	0.2	F	RI, MS
45	1643	(E)-2-Decenal	003913-81-3	287.1	5.6	30.5	0.7	123.3	2.7	Std	RI, MS, Std
46	1656	1-Nonanol	000143-08-8	36.5	0.7	9.3	0.2	34.8	0.7	F	RI, MS
47	1718	α-Selinene	000473-13-2	0.0	0.0	0.0	0.0	38.0	0.6	A	RI, MS
48	1748	α-Farnesene	000502-61-4	493.0	6.9	618.1	10.2	803.4	12.9	A	RI, MS
49	1758	(E,Z)-2,4-Decadienal	025152-83-4	99.6	1.6	25.5	0.5	38.0	0.7	D	RI, MS
50	1772	Methyl salicylate	000119-36-8	35.8	0.3	30.2	0.3	0.0	0.0	I	RI, MS
51	1810	(E,E)-2,4-Decadienal	025152-84-5	141.7	2.2	25.7	0.5	57.6	1.0	Std	RI, MS, Std
52	1837	Hexanoic acid	000142-62-1	65.9	1.6	15.8	0.4	37.8	1.0	Std	RI, MS, Std
53	1848	(E,E)-Farnesol	000106-28-5	0.0	0.0	25.8	0.3	0.0	0.0	K	RI, MS
54	1853	Nerylacetone	003879-26-3	58.7	0.6	0.0	0.0	0.0	0.0	M	RI, MS
55	1871	Benzyl alcohol	000100-51-6	72.3	1.0	0.0	0.0	87.5	1.4	J	RI, MS
56	1908	Phenylethyl alcohol	000060-12-8	132.4	1.8	60.4	1.0	161.6	2.5	J	RI, MS

Supplementary Table 3. (*continued*)

No.	RI	Volatile compound	CAS	Hojiblanca		Mission		Lucca		Quantification[b]	Identification[c]
				Peak area[a]	μg/200 μL	Peak area	μg/200 μL	Peak area	μg/200 μL		
57	1939	2-Ethylhexanoic acid	000149-57-5	20.4	0.5	0.0	0.0	0.0	0.0	E	RI, MS
58	1942	(E)-3-Hexenoic acid	001577-18-0	29.6	0.7	0.0	0.0	0.0	0.0	E	RI, MS
59	1959	(E)-2-Hexenoic acid	013419-69-7	243.4	5.7	0.0	0.0	0.0	0.0	E	RI, MS
60	1960	1-Dodecanol	000112-53-8	41.1	0.3	0.0	0.0	0.0	0.0	K	RI, MS
61	1969	δ-Octalactone	000698-76-0	7.7	0.1	7.2	0.1	0.0	0.0	Std	RI, MS, Std
62	1995	Phenol	000108-95-2	6.6	0.1	0.0	0.0	0.0	0.0	J	RI, MS
63	2019	Diphenyl ether	000101-84-8	6.3	0.1	0.0	0.0	0.0	0.0	J	RI, MS
64	2036	(E)-Nerolidol	040716-66-3	194.7	1.6	201.8	2.0	0.0	0.0	K	RI, MS
65	2052	Octanoic acid	000124-07-2	29.7	0.5	39.7	0.8	18.4	0.4	Std	RI, MS, Std
66	2056	Dimethyl salicylate	000606-45-1	29.8	0.3	33.2	0.4	0.0	0.0	I	RI, MS
67	2076	Elemol	000639-99-6	0.0	0.0	17.5	0.3	0.0	0.0	J	RI, MS
68	2153	Nonanoic acid	000112-05-0	49.8	0.6	53.6	0.7	44.7	0.6	Std	RI, MS, Std
69	2157	3,5-Dimethylphenol	000108-68-9	0.0	0.0	29.4	0.5	26.6	0.4	J	RI, MS
70	2172	4-Ethylphenol	000123-07-9	48.2	0.7	0.0	0.0	0.0	0.0	J	RI, MS
71	2198	δ-Decalactone	000705-86-2	0.0	0.0	25.8	0.9	22.1	0.8	Std	RI, MS, Std
72	2216	Methyl palmitate	000112-39-0	8.6	0.1	0.0	0.0	0.0	0.0	I	RI, MS
73	2246	α-Cadinol	000481-34-5	78.3	1.1	42.0	0.7	22.2	0.3	J	RI, MS
74	2317	Geranic acid	000459-80-3	0.0	0.0	0.0	0.0	15.8	0.3	N	RI, MS
75	2371	Dihydroactinidiolide	015356-74-8	24.3	0.3	0.0	0.0	0.0	0.0	J	RI, MS
76	2380	Coumaran	000496-16-2	48.8	0.7	0.0	0.0	0.0	0.0	J	RI, MS
77	2399	Methyl jasmonate	001211-29-6	30.2	0.3	0.0	0.0	0.0	0.0	I	RI, MS
78	2570	Vanillin	000121-33-5	105.3	2.6	49.4	1.5	58.3	1.7	H	RI, MS
79	2656	δ-Tetradecalactone	002721-22-4	0.0	0.0	49.5	0.9	35.8	0.6	Std	RI, MS, Std
80	2900	Hexadecanoic acid	000057-10-3	37.7	0.3	0.0	0.0	0.0	0.0	Std	RI, MS, Std
Total				8034.0	145.1	4652.3	104.1	9567.9	231.9		

[a] $\times 10^5$. [b] Quantification: Std, authentic standard; A, toluene; B, 2-hexanone; C, hexyl acetate; D, 2,4-decadienal; E, hexanoic acid; F, 2-hexanol; G, *trans*-2-hexenal; H, *trans*-2-heptenal; I, ethyl decanoate; J, 2-phenoxyethanol; K, 1-hexadecanol; L, nonanal; M, 2-undecanone; N, decanoic acid. [c] Identification: RI, retention index; MS, mass spectral fragmentation pattern; Std, authentic standard. Each experiment was performed once.

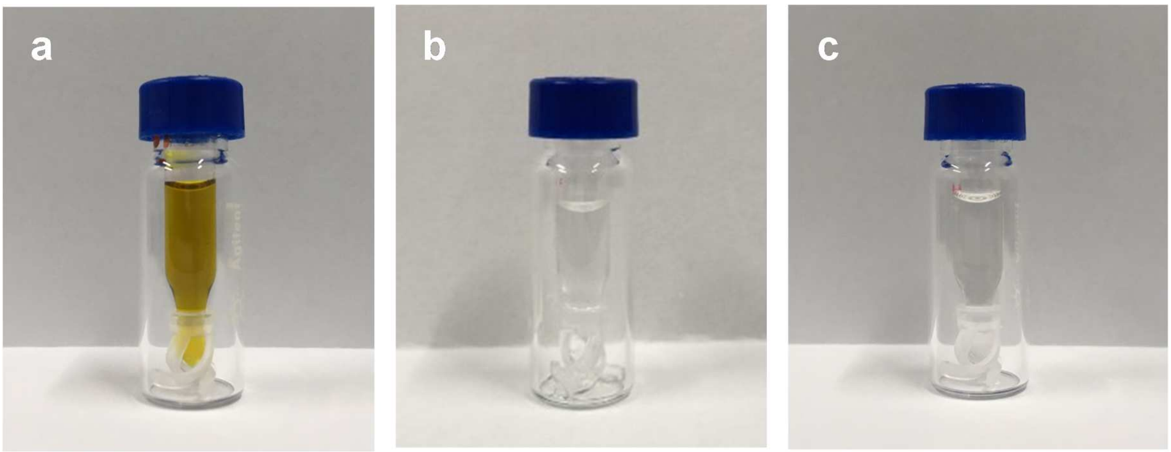

Supplementary Fig. 1 Aromatic extracts obtained by OA-LLE, SAFE, and OA-LLE + SAFE. The OA-LLE extract from EVOO (**a**) contained non-volatiles such as pigments that were dark green. The extracts obtained by SAFE (**b**) and OA-LLE + SAFE (**c**) were colorless and transparent because the non-volatiles were removed by distillation.
